# Prevention after Cure

**Published:** 1920-05-29

**Authors:** 


### The Hospital

The Workers' Newspaper of Administrative Medicine and Institutional Life, Administration, National Insurance and Health.

No. 1773, Vol. LXVIII. SATURDAY, MAY 29, 1920. PRICE TWOPENCE

### PREVENTION AFTER CURE.

It is not readily possible to see that the apparently imminent action of the Ministry of Health in burdening the rates still further with institutional upkeep is a necessarily retrograde step. It was with surprise that we noted this view expressed in a famous daily contemporary, which argued on the lines that in the greater hospitals there is nowadays a gross preponderance of preventable disease under treatment, and that therefore the most obviously correct policy is to devote our main strength to developing those phases of medicine and its administration which are essentially preventive, instead of, in the first place, extending existing curative establishments. In its main outline such a scheme is reasonably justified by inspection of past case records of most of our hospitals, but the urging of its immediate and full application appears to us, at the present moment, to record something of an extremist's view of the situation.

Very rightly *The Times* medical correspondent has for months past insisted upon public realisation of the amazing amount of sickness and disease which should never have been allowed to occur among the people, and that it is absolutely essential for the profession to devote more attention to the so-called "beginnings of disease." Nevertheless, it is a very daring reformer who claims that at the moment the country is circumstantially prepared for a complete reversion from the institutional methods which have served—and served by no means badly—for so many years past. We do not speak in any ultra-conservative spirit, but feel that, whatever views are held as to the true ideal and the possibilities of creating a medical Utopia in Great Britain, it is in any case essential to realise that we are in 1920 only at the verge of a great transition period.

In years to come the country will undoubtedly have much cause to be thankful to those who first appreciated the possibilities of organised prevention—and indeed to those who are unremitting in their efforts to recount these principles where the public may both learn and understand them. Still, for the time being, progress is not only bound to be slow, but if its rapid acceleration requires that the immediate relief of the present hospital financial crisis should be in any degree shelved, then we feel that it had better be woven into our policy as a guiding principle rather than a drastic institutional reform.

Let us turn also to the view which the average layman must take of the matters as they affect him personally. If the Press is to educate the public on medical subjects, it must remember that in them, above all others, the non-professional reader at once tends to apply their particular moral to himself, and it will undoubtedly take many generations of both laymen and doctors before either can act ideally upon the true preventive principle. We must at all costs not confuse those we attempt to teach. For many years people must and will appeal more frequently to the doctor to cure them than to prevent them from becoming ill. Also it is a bold administrator who would deny a person's right to treatment because he never need have become ill! What is needed is gradual development and tightening of a bond of sympathy and understanding between the people and the medical services, public or private, of the country. In this way only will true prevention in the end be practised.

On the other hand, one channel at least exists whereby both ends may be simultaneously served. There are enormous possibilities for local enterprise, possibly State aided, forwarding at the same time both small institutional and—we may perhaps use the term—"clinic" development on the part of general practitioners themselves, which can in no way interfere with, but rather aids, the work of the greater hospitals. Such organised units might, in fact, markedly relieve congestion in the wards of the central hospitals, and *The Times* aptly cites a recent instance where, "in a small country town of 10,000 inhabitants a small cottage hospital—corresponding in many ways to the clinic at St. Andrew's—has been opened, and the general practitioners of the town have staffed it. . . . There cases are investigated and treated, which in earlier days went to crowd out the city hospitals some twenty miles distant." Certainly there are great possibilities for the small local unit to play its part in the country's medical economy, but nevertheless all such schemes must bear fruit gradually. The basis on which they all must rest, and the vital centres of the medical world must lie in the greater, are the perfectly equipped hospitals of our cities, with their staffs drawn from the finest of the professional world, and it is our plea that whatever the ultimate ramification of the Ministry's activities, the first and foremost problem with which the country is concerned is the placing of its greater hospitals on a sound and satisfactory financial footing.

